# SurgeCon: Priming a Community Emergency Department for Patient Flow Management

**DOI:** 10.5811/westjem.2019.5.42027

**Published:** 2019-07-05

**Authors:** Christopher Patey, Paul Norman, Mehdee Araee, Shabnam Asghari, Thomas Heeley, Sarah Boyd, Oliver Hurley, Kris Aubrey-Bassler

**Affiliations:** * Memorial University of Newfoundland, Discipline of Family Medicine, St. John’s, Newfoundland; † Eastern Health, Carbonear Institute for Rural Research and Innovation by the Sea, Carbonear General Hospital, Carbonear, Newfoundland

## Abstract

**Introduction:**

Canadian emergency departments (ED) are struggling to provide timely emergency care. Very few studies have assessed attempts to improve ED patient flow in the rural context. We assessed the impact of SurgeCon, an ED patient-management protocol, on total patient visits, patients who left without being seen (LWBS), length of stay for departed patients (LOSDep), and physician initial assessment time (PIA) in a rural community hospital ED.

**Methods:**

We implemented a set of commonly used methods for increasing ED efficiency with an innovative approach over 45 months. Our intervention involved seven parts comprised of an external review, Lean training, fast track implementation, patient-centeredness approach, door-to-doctor approach, performance reporting, and an action-based surge capacity protocol. We measured key performance indicators including total patient visits (count), PIA (minutes), LWBS (percentage), and LOSDep (minutes) before and after the SurgeCon intervention. We also performed an interrupted time series (ITS) analysis.

**Results:**

During the study period, 80,709 people visited the ED. PIA decreased from 104.3 (±9.9) minutes to 42.2 (±8.1) minutes, LOSDep decreased from 199.4 (±16.8) minutes to 134.4(±14.5) minutes, and LWBS decreased from 12.1% (±2.2) to 4.6% (±1.7) despite a 25.7% increase in patient volume between pre-intervention and post-intervention stages. The ITS analysis revealed a significant level change in PIA – 19.8 minutes (p<0.01), and LWBS – 3.8% (0.02), respectively. The change over time decreased by 2.7 minutes/month (p< 0.001), 3.0 minutes/month (p<0.001) and 0.4%/month (p<0.001) for PIA, LOSDep, and LWBS, after the intervention.

**Conclusion:**

SurgeCon improved the key wait-time metrics in a rural ED in a country where average wait times continue to rise. The SurgeCon platform has the potential to improve ED efficiency in community hospitals with limited resources.

## INTRODUCTION

Emergency department (ED) crowding is a perennial Canadian healthcare concern.^1^ Misdiagnosis, declining patient confidentiality and satisfaction, and bed-block (when all available beds are occupied and patients are left in corridors and ambulances) are only some of the resulting issues.^1^,^2^ Amidst a perfect storm of recent Canadian Association of Emergency Physician national targets,^1^ increasing financial and resource pressures,^3^ loss of full-care community providers,^4^ and aging populations,^5^ Canadian EDs are grappling with some of the longest wait times compared to peer industrialized countries.^6^ The Newfoundland and Labrador (NL) Department of Health and Community Services has referred to EDs as the “front door” to the province’s healthcare system.^7^ After missing its own wait time benchmarks in 2016,^8^ NL’s Eastern Regional Health Authority joined the chorus of emergency care providers across Canada hunting for a solution to crowded ED care.^7^,^9^–^13^ Large-scale process improvement is effective at urban facilities,^14^,^15^ but NL is predominately rural^16^ with many small EDs facing crowding,^17^ increasing hospital occupancy rates,^18^ and restrictive department sizes.^19^

A key problem with the literature related to ED quality improvement is the focus on urban tertiary/quaternary centers rather than smaller, rural EDs where the factors affecting patient flow may be different. In many rural communities, the ED is often the first and only choice to receive care.^20^,^21^ Our team from the rural NL community of Carbonear created SurgeCon as a way to counteract these challenges. Rural EDs are an ideal setting to implement innovative models since they are more agile and have the potential to improve healthcare delivery and patient outcomes for a considerable portion of the population.^22^ The ED in Carbonear represents a new frontier for the assessment of ED quality improvement interventions given the size of the communities it serves and the resources at its disposal, while facing the same challenges as larger centers.

SurgeCon is a pragmatic, ED management platform that includes a series of interventions acting together to improve ED efficiency and patient satisfaction. More specifically, the interventions target three key areas: 1) ED organization and workflow; 2) action-based ED management, and 3) the establishment of a patient-centric environment. SurgeCon is a term derived from the concepts of patient surge and defense readiness condition (DEFCON), which is a military escalation system. It is explicitly designed to address ED crowding by implementing commonly used methods for increasing ED efficiency in concert with technological innovation. The 45-month, proof-of-concept investigation described in this paper assessed the impact of SurgeCon on key, patient-flow and wait-time indicators in a rural community ED strained by a large volume of lower acuity patients. Specifically, we examined 1) time until physician initial assessment (PIA), 2) the proportion of patients who registered but left without being seen by a physician or his/her delegate (LWBS), and 3) mean length of stay for departed patients (LOSDep).

## METHODS

### Study Design and Time Period

We used a quasi-experimental research design to assess the impact of the SurgeCon intervention. The data used in this study considered a period of 15 months before the intervention (July 1, 2013, to September 30, 2014, inclusive) and 30 months after the intervention (October 1, 2014, to March 31, 2017, inclusive).

**Population Health Research Capsule**

What do we already know about this issue?

Emergency department (ED) wait times continue to rise annually in Canada with a significant increase in crowding and cost to the healthcare system.

What was the research question?

How does the new initiative “SurgeCon” impact patient flow and wait times in a rural community ED?

What was the major finding of the study?

SurgeCon resulted in significant improvements in key wait-time metrics in a rural community ED.

How does this improve population health?

Decreasing wait times and crowding in the ED increases quality of care and improves patient health outcomes.

### Study Setting

Carbonear General Hospital is an 80-bed hospital located on the southeastern coast of Newfoundland, the island portion of Canada’s easternmost province Newfoundland and Labrador.^23^ Carbonear is located approximately 75 minutes (~100 kilometers) from the only two provincial, tertiary referral hospitals, which are located in the capital city of St. John’s. The community of Carbonear has a population of approximately 5,000, and the Carbonear Hospital provides services to a catchment population of approximately 40,000.^24^ There are four full-time and four part-time emergency physicians (EP), one full-time nurse practitioner, two dedicated paramedics, and a maximum of three nurses at one time allocated to the ED.

### Data Collection and Integration

Routinely collected data provided by the Eastern Regional Health Authority included patient ED wait times from the point of registration to patient departure from the ED (eg, discharge, hospital admission). We used IBM Cognos Analytics (Armonk, New York), a business intelligence analytics platform, to provide monthly reviews from July 1, 2013, to March 31, 2017. A 45-month retrospective review of ED patient flow metrics was completed and compiled in May 2017. Values for each of these metrics were captured each month for 15 months prior to initiation, 15 months in the early intervention period, and 15 months in the late intervention period. This quality improvement initiative was conducted exclusively for ED improvement purposes and did not require ethics approval from the provincial Health Research Ethics Authority.

### Intervention

Our intervention is composed of seven parts, which were implemented sequentially as described below.

1. *Independent External Review:* In October 2014 we contracted an ED quality improvement (QI) consulting firm to conduct a comprehensive assessment of the organization and function of the Carbonear ED. This review formally quantified performance, clarified key improvement issues, and prepared staff members to begin the improvement process.
2. *Lean*^25^: Studies indicate that Lean strategies have been associated with improved ED performance and patient satisfaction.^26^,^27^ Frontline staff attended two days of Lean training to facilitate the implementation of improvement initiatives and encourage ED staff to become active participants in the improvement process. The course was formulated to directly improve the flow of patients at the frontline of operational EDs by using simulation exercises, innovative surge-management software tools, and inventive approaches to real-life ED flow problems.
3. *Rapid Assessment “Fast Track” Zone:* Fast-track areas staffed by midlevel providers can improve patient flow and reduce ED crowding, wait times, LOSDep, and LWBS rates without affecting quality of care.^28^–^32^ We created a rapid assessment zone by re-designating an underused waiting area adjacent to the ED triage area, enabling a 20% increase in physical ED space. Adding two new short examination tables and a chair in this area and using it for patient assessment, blood tests, electrocardiograms, and other minor procedures, maximized accessibility and ambulatory patient throughput. By removing competition for assessment space, this area also doubled for early reassessments and discharges, and as an independent nurse practitioner area.
4. *Patient Centeredness:* ED staff often believe that lower acuity patients should not seek care in an ED setting while also distinguishing patients based on “their legitimacy to be treated within the ED.”^33^ This belief system can create a culture of apathy and disregard toward patients who require primary and non-urgent care. Based on evidence that promoting a culture of patient “worthiness” improves patient satisfaction and ED efficiency,^33^,^34^ we initiated multiple 30-minute staff educational sessions reinforcing the following three main topics: 1) providing quality ED care to all patients regardless of urgency; (2) treating all patients with respect; and (3) always considering the patient’s visit to an ED to be necessary as they may have no other option. We also provided strategies to get the patient to the provider in a timelier manner (eg, physicians going to triage, moving patients from clinical assessment spaces back to waiting room/alternate waiting room, faster admitted patient extraction, efficient use of fast track areas, etc.). Patient-centeredness was also addressed through improving the ED environment, as a patient’s waiting environment is a better predictor of patient satisfaction than wait times.^35^ We removed all wall postings not pertinent to ED staff and patients, and all subsequent postings required departmental approval before being placed in a central location. We also redecorated the ED waiting room and patient examination rooms with framed photographs of coastal communities from the hospital’s rural catchment area.
5. *Physician Initial Assessment “Door to Doctor” Focus:* A number of studies have found a strong correlation between patient satisfaction and PIA; the shorter the PIA, the more satisfied the patient.^36^–^38^ To reduce the time to PIA we used the following strategies
  - ED staff briefly assessed patients even when formal assessment space was not immediately available.
  - EPs were provided with the option of triaging with nursing staff with the potential goal of patient discharge directly from the triage room without waiting.
  - Triage nurse-driven orders (eg, symptom management, laboratory testing, diagnostic imaging, etc.) were only applied on patients who would be waiting longer than one hour to see a physician. If the patient could have been seen by a physician within an hour, waiting for potentially unnecessary test results could delay the PIA.
  - If there were no available beds, EPs assessed patients arriving on ambulance stretchers in the hallway to initiate investigations instead of waiting for a bed to be free.
  - Staff attempted to offer short physician assessments prior to ordering diagnostic tests that could have potentially delayed discharge.
6. *Performance Data and Patient Flow*: Regular performance reporting enhances ED functioning and assists with improvement strategies.^27^,^38^ Previously, the Carbonear EPs, nurses, managers, and staff infrequently reviewed ED performance data; however, as part of SurgeCon, our team circulated and clearly posted data in a prominent area of the department on a monthly basis. Individual physicians were informed of their monthly PIA times compared to the ED average.
7. *Action-based Surge Capacity Protocol:* We created and implemented a unique frontline, action-based tool that helps ED staff (paramedics, nurses and physicians) manage their actions to actively reduce patient surges and wait times and increase patients’ access to emergency medical care. This tool uses algorithms to prompt the appropriate and timely use of volume-based staffing and management and overcapacity protocols, which may otherwise be overlooked by distracted frontline ED staff. When the ED is at overcapacity, ED staff require additional external support and resources, which are obtained by calling management. The prescribed actions included in the protocol when patient demand exceeds capacity in an optimal flow environment (eg, SurgeCon 4 & 5) are designed to find ways to remediate systemic issues that exist outside the ED and require managerial-level interventions that contribute to holistic operating conditions. This protocol converts key performance indicators into instructions for ED providers using a three-step process:
  - An EP, nurse, or administrator enters counts of specific indicators every two hours (eg, number of beds available, number of admitted patients, number of patients not seen) onto a whiteboard as part of their regular workflow.
  - As a result of step (i), the team gains awareness of workload that can be shared with key stakeholders both internal and external to the ED. External stakeholders could determine bed availability, extract patients from the ED to an assigned inpatient bed, temporarily increase nurse and physician staffing, contact admitting consultants, and contact primary care paramedics for assistance.
  - Using the visual board, the team adds the scores to get a total. This total score falls in one of five graduated levels, each with a set of prescribed actions. For example, a total score of 40 or more is level 5, with associated actions such as “Send all lower acuity patients to the waiting room.” This scoring algorithm provides clarity for frontline staff in real time (Appendix II). Moving stable patients or visitors from clinical assessment spaces back to a primary or alternate waiting room is in line with our objective of creating a patient-centered environment as this allows for more non-assessed patients to become the center of care.^39^,^40^

### Outcome Measures

The ED team (EP, nurses, and nursing management) manually collected data for PIA, LOSDep, and LWBS from Carbonear’s hospital records, and reviewed it monthly from July 1, 2013–March 31, 2017. In May 2017, we retrospectively reviewed PIA, LOSDep, and LWBS in each study period and compiled the data for analysis.

An ED team (clinical manager, site clinical physician chief, nurse practitioner, nursing-appointed chairperson, and various other frontline staff) identified the following as outcome measures before the intervention began:

- PIA: mean time (in minutes) from patient triage to first assessment by a physician or their delegate (nurse practitioner, trainee, etc.). PIA is also referred to as “arrival to provider” or “door to doctor.”^1^,^41^,^43^
- LWBS: percentage of patients who leave the ED without an assessment by a physician or their delegate. LWBS is also referred to as “left before being seen.”^1^,^43^,^44^
- LOSDep: mean time interval (in minutes) between patient being triaged and discharged from the ED (in minutes). LOSDep is also referred to as “ED length of stay for discharged patient.”^1^,^43^

Senior ED management send out scorecards with PIA, LOSDep, and LWBS data to local ED management for their review. To get PIA and LOSDep times, they take the earliest of three time stamps (arrival time, triage time, or registration time) and use that as the patient’s time of arrival.

### Data Analysis

Interrupted time series (ITS) analysis is an effective statistical approach to assess the impact of an intervention in a quasi-experimental research design.^45^ To design a robust ITS analysis, we used guidelines introduced by Bernal, Cummins, and Gasparrini,^46^ and selected a single-group, segmented time-series regression model. Time series analyses calculate the change in an outcome over time before an intervention is introduced, and then assess the immediate (month after introduction) and long-term (change in trend over time) effects of the intervention after adjusting for this pre-intervention trend. Thus, an immediate effect is significant if there is a statistically significant change in outcome in the month after program introduction from what would have been expected if the pre-intervention trend had continued. The long-term effect of the intervention is assessed by determining if there is a difference between the rates of change in outcome over time (slope) from the pre- to post-intervention periods.

For the purpose of this study, we initially conducted a segmented time-series regression model with two segments (pre-intervention and post-intervention) to identify whether there was any significant change in outcomes after implementation of the intervention. Then, we graphed the data and visualized two breakpoints occurring after the intervention. Therefore, we used a three-segment ITS model to more accurately represent our data. To identify the optimum breakpoints, we visually estimated that the breakpoints would be somewhere between months 15 and 30 of our 45-month study period. We looked for any sign changes and big swings in the values of the estimated coefficients as well as the model fitness criterion.

The analyses suggested that the estimates of the early intervention become significant after six months of the intervention and remained so until 15 months of intervention with no variation in the magnitude or direction of the estimated coefficients. The analyses also suggested that the estimates of the late intervention become significant after 15 months. Increasing the duration of the late intervention period did not show any significant change in model fitness, magnitude and direction of the estimated coefficients of the late intervention until 30 months after initiation of the intervention. For this reason, the three-segmented linear regression model with segments including before intervention (July 1, 2013 to September 30, 2014, inclusive), early intervention (October 1, 2014 to December 31, 2015, inclusive), and late intervention (January 1, 2016 to March 31, 2017, inclusive) have been fitted to the data.

Finally, we added the number of visits per month as a covariate in the model to reassess the model fitness and any statistically significant changes in the estimated coefficients. We also conducted seasonality analysis to see whether the data experienced regular and predictable changes, and found no periodic fluctuations in all calendar seasons. Details about the parameters in the model are available in Appendix I. We used Stata version 14.2 (StataCorp, College Station, Texas) for statistical analyses.

## RESULTS

Over the entire study period, there were 80,709 patient visits to the Carbonear ED. Table 1 shows the characteristics of ED visits.

Overall, in this 45-month study PIA decreased from a mean of 104.3 minutes (±9.9 standard deviations) to 42.2 (±8.1) minutes, LOSDep decreased from 199.4 (±16.8) minutes to 134.4(±14.5) minutes, and LWBS decreased from 12.1% (±2.2) to 4.6% (±1.7). The results of a segmented time series analysis are as follows:

### Physician Initial Assessment

As described in Table 2, the ITS regression with two segments shows an immediate effect (level change) of −19.8 minutes (p<0.01; 95% confidence interval [CI], −33.68 to −5.89]) drop in PIA and a long-term effect (slope change) of −2.72 (p< 0.001; 95% CI, −3.97 to −1.48) after the intervention.

**Figure 1:**
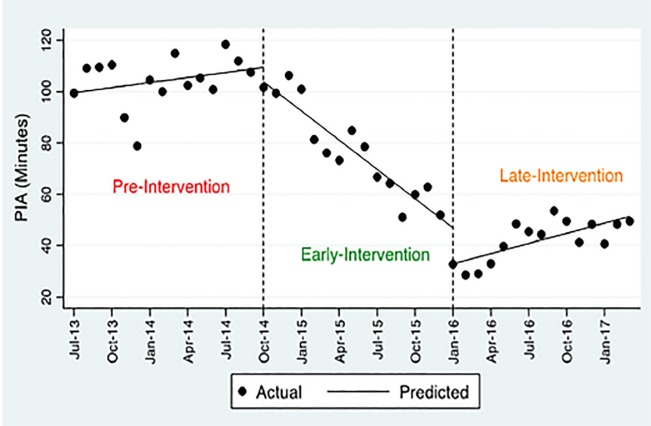
Physician Initial Assessmen (PIA). Visual depiction of the overall declining trend in time to physician initial assessment over entire post-intervention period.

Using the three-segment model, the level change shows a reduction in both early and late intervention by −5.59 (p=0.186; 95% CI, −13.99 to −2.81) and −13.99 (p<0.004; 95% CI, −23.35 to −4.63), respectively. The change in PIA slope was mainly due to changes in the early intervention period where the PIA significantly decreased every month by −4.45 minutes on average (p<0.001; 95% CI, −5.59 to −3.32). A monthly increase of 5.12 minutes (slope change) in PIA can be seen during the late-intervention period (p<0.001; 95% CI, 4.19 to 6.05) compared to early intervention. However, considering the linear trend of −3.80 (p< 0.001; 95% CI, −4.45 to −3.15) in the early intervention vs l.3 (p< 0.001; 95% CI, 0.60 to 2.05) in the late intervention and the level change of 13.99 (p<0.004; 95% CI, −23.35 to −4.63) in the late intervention, there was an overall decline over the entire post-intervention period. This can be verified upon visual inspection of Figure 1.

### Length of Stay Until Departure from Emergency Department

According to the two-segment model, the immediate effect of the intervention was a 17.5-minute decrease in LOS for departed patients, but this difference is not statistically significant (p<0.150). The long-term effect (slope change) on LOSDep is statistically significant (p<0.002) with a reduction of three minutes per month after implementation of the intervention.

**Figure 2:**
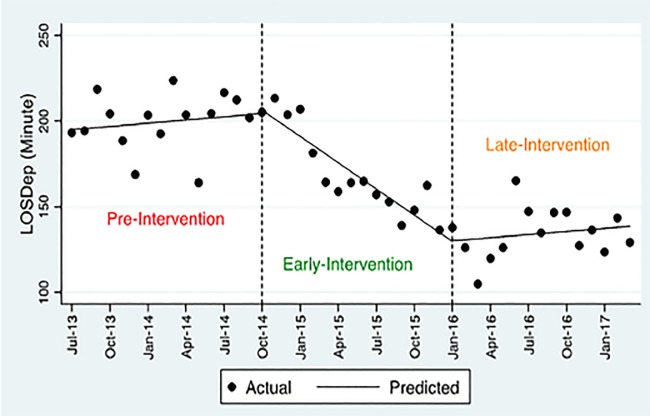
Length of stay for departed patients (LOSDep). Visual depiction of the overall diminishing trend during the entire post-intervention period.

The three-segment model reports a statistically non-significant level change in both early intervention (1.93, p=0.82) and the late intervention period (0.43, p=0.97) (Table 3). It also reveals a significant decrease of 5.7 minutes in LOSDep (slope change) during the early period (p<0.001) and 5.7 minutes increase (slope change) in the late period (p<0.001). By looking at the linear trend results for the early intervention period (−5.1, p<0.001, 95% CI, −6.64 to −3.59) and late intervention period (0.6, p=0.43, 95% CI, −0.92 to 2.12), the overall diminishing trend seems to be preserved during the post-intervention period (−3, p<0.002; 95% CI, −4.87 to −1.14). This also can be verified upon visual inspection of Figure 2.

### Left Without Being Seen

**Figure 3:**
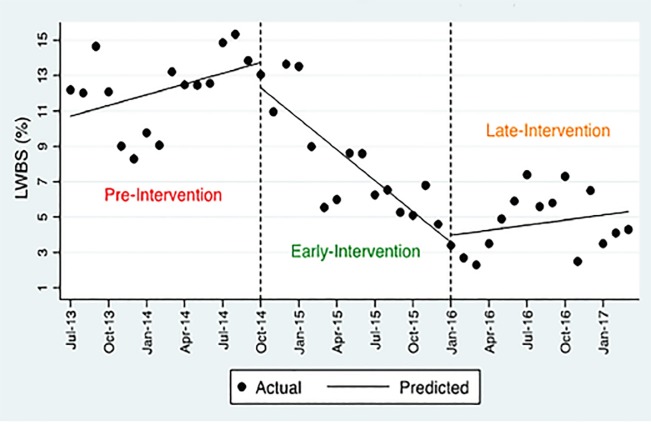
Patients left without being seen (LWBS). Visual depiction showing overall long-term trend diminshing over the post-intervention period.

Applying a two-segment model shows an immediate effect of 3.8% decrease (p<0.02; 95% CI, −6.87 to −0.75) and the long-term effect of decrease by 0.4 % (p<0.004; 95% CI, −0.73 to −0.15) on LWBS after the implementation of the intervention. Using the three-segment model, a statistically non-significant level change of −1.42% (p=0.340) in the early period and 0.41% (p=0.718) in the late period is seen (Table 3). This model also shows a drop in the long-term effect of 0.78% (p<0.001; 95% CI, −1.14 to −0.43) and then an increase of 0.68% during the late intervention (p<0.001; 95% CI, 0.41 to 0.95). The linear trend in the early intervention period shows a significant decrease by 0.58 (p<0.001; 95% CI, −077 to −0.40) and a non-significant trend of 0.09 (p=0.38). Since the overall slope change is declining (Table 2), the slope of linear trend in the early-intervention period is decreasing by 0.58 (p<0.001) and the positive slope of linear trend is not statistically significant (0.09, P=0.38), the overall long-term effect (trend) is diminishing over the post-intervention period (Figure 3).

To control for the effect of patient volume, we adjusted the model by adding number of visits per month (“Visits”) to the three-segment model. The results (Table 4) show visits to be associated with PIA (0.02, 95% CI, 0.00 to 0.04; p<0.05) and LWBS (0.01, 95% CI, 0.00 to 0.01; p<0.04).

Overall, the adjusted model (Table 4) is consistent with the primary model (Table 3). No abnormal changes in the direction/sign of coefficients were seen, except the magnitude of coefficients, which have partially changed between the early- and late-intervention periods. Comparing the primary and adjusted models, the only difference is that the baseline slopes become statistically significant in PIA ([0.66, p=0.17] vs ([0.76, p<0.03]) and LWBS ([0.20, p=017] vs. [0.23, p<0.03]), respectively.

## DISCUSSION

Before implementing SurgeCon, there was anecdotal evidence that the Carbonear ED was not achieving national benchmarks and had the highest LWBS rate relative to similar-sized EDs in NL.^8^ This study provides evidence that EDs can be adapted to efficiently provide urgent and non-urgent care in rural communities. All of our analyses showed an upward trend (ie, worsening) in outcomes over time in the pre-intervention period. After the implementation of the SurgeCon platform at the Carbonear ED, all outcomes showed a significant improvement. While the trend change was reversed in the late-intervention period, the rate of change was either non-significant or slower compared to immediate and long-term effects of the intervention in the early intervention period. The worsening trend from the early-to late-intervention period is likely a combination of an increase in ED volume and sustaining the gains long term. A refresher session may improve the results after the first 15 months. Sensitivity analyses did not show any significant change in the model’s fitness or estimated coefficients where we applied the three-segment models to different subsets of the data. Moreover, repeating these processes by adding the variable number of visits per month did not show any significant change in the model’s fitness or estimated coefficients.

It is worth highlighting that the dramatic improvements in outcomes demonstrated here occurred despite a 25.7% increase in patient visits from the pre- to the late-intervention periods. Moreover, no additional staff were hired during the study period. The increased volume were predominantly patients who were categorized as Canadian Triage and Acuity Scale (CTAS) 4 or non-urgent visits, which are often considered to be those that are amenable to treatment in primary care. The ability to provide care to a larger volume of patients without increasing wait times may be due in part to improved team awareness. The mechanisms that lead to improved “team awareness” in the context of the SurgeCon platform are a result of the tasks and goals set by the protocol. The protocol addresses issues related to harmful assumptions, establishes a common decision-making process, improves communication, and sets expectations for everyone on the ED team through role assignment. This is achieved via the protocol by defining the problem, the strategies to overcome them, and the overall goals of the department depending on the level of demand at any given time.

Our study does not suggest EDs can replace traditional means of accessing primary care; however, they can be relied upon as a secondary alternative approach to providing primary care in communities where access to a family physician may be challenging. In the community surrounding the Carbonear Hospital there has been a large loss of primary care physicians who retired in recent years. Recent studies have found evidence that rural patients are more likely to use EDs for non-urgent reasons when compared to their urban counterparts.^47^,^48^ Geographic proximity to EDs and the likelihood of being seen by a regular family physician were found to be important factors influencing this discrepancy.^48^–^50^

In recent years many studies have evaluated Lean initiatives,^51^–^53^ fast-track areas in the ED,^54^–^6^, physicians in triage,^57^ and full capacity protocols,^58^ although the vast majority of these studies took place in urban centers. Furthermore, most studies examine just one initiative while our study included a large initiative with seven parts. The biggest strength to this study is that the initiatives were developed and implemented by a team of frontline practitioners (physicians and registered nurses) who have experienced firsthand the inefficiencies of the ED.

## LIMITATIONS

There are a few limitations that should be taken into consideration when interpreting the findings from this study. First, the generalizability of our results is limited given that implementation occurred at a single site. That said, the nature of a proof-of-concept initiative is to test a novel process on a small scale for feasibility and impact. The scoring system as shown in Appendix II also has limited generalizability because it is not normalized against ED size, and gives exact numbers (e.g., number of occupied beds) instead of proportions of beds that are full. Another limitation encountered during the study is particular to the hospital setting, where we could only call the inpatient unit for the immediate extraction from the ED during SurgeCon 5, instead of proactively calling. This was due to a negotiation between busy units, when ideally the unit would be called prior to this level of overcapacity.

Second, once patients had been seen by ED staff they were sorted using the CTAS. This triaging was not considered in our analysis because for this QI initiative, we used data aggregated on a monthly basis. We did not have data at the individual level to assess the associations with CTAS. Visits based on CTAS shows slight fluctuations in CTAS I and II during the study period and increasing number of visits with CTAS III and IV. The percentage of unclassified CTAS patients was also higher in the pre-intervention period than in the post-intervention period. Third, we did not measure 72-hour return to ED rate; however, a 2016 study by Cheng et al.^59^ found that this often-cited measure is not reliably indicative of ED quality. Another potential limitation is that we only used 45 time points and it is known that power in ITS analyses increases with a larger number of time points.^46^ The decision to use mean instead of median might be viewed as a limitation within the context of this study. Although median may perform better for a skewed distribution such as length of stay in hospital and wherever the goal is to represent a typical length of stay, mean is more sensitive to magnitude and is a more representative statistic from the point of view of assessing health system costs and efficiency.^60^–^62^

One may question the decision to use segmented regression instead of autoregressive integrated moving average (ARIMA).^63^ Although ARIMA models inherently account for autocorrelation, non-stationarity and seasonality, they require a sufficient number of data points and observations in the pre- and post-intervention periods (a minimum of nine data points and over 100 observations).^64^ Segmented regression on the other hand is one of the most common interrupted time series methods used in health sciences research.^64^ It is similar to linear regression and is suggested for functions that cut segments of time particularly for studies such as the one described here where the points of switching segments are known. They are also more flexible for multivariate analysis.^46^,^65^ To ensure elements covered through ARIMA models were included in our analysis, we checked for autocorrelation, non-stationarity, and seasonality before running the model (Appendix). With this in mind, due to the flexibility and applicability in the context of a proof of concept study we conducted a segmented regression analysis.^66^

As there is a trend toward increasing PIA, LOSDep, and LWBS between the early- and late- intervention stages, it is possible some of the measures may have returned to pre-intervention levels if the study had continued for longer. It is possible that a “refresher” training session may be needed to combat this. It is also possible that a decrease in LWBS will result in a slight increase in the number of patients leaving before treatment is completed as some patients will not want to wait for test results regardless of how quickly the doctor sees them. Unfortunately, the routinely collected data used in this study did not include the number of patients who left before completing treatment. Another possible limitation of this study is the Hawthorne effect in which individuals behave differently when they know they are being observed. This may have led to ED staff modifying their behavior over the course of this study. Finally, physicians and nurse practitioners manually entered PIA, which may have impacted data quality.

## CONCLUSION

Our team recognized the necessity of a hospital-wide response from the outset, and designed and implemented SurgeCon accordingly. We took careful stock of existing resources in the ED and developed the comprehensive SurgeCon strategy around them. This was achieved by aligning the ED team around performance gains and approaching other key stakeholders in the system to help with output issues. This study provides evidence that interventions such as SurgeCon can result in significant gains with regard to key wait-time metrics in a rural community hospital with limited resources.

## Supplementary Information





## Figures and Tables

**Table 1:** Characteristics of patient visits to the Carbonear emergency department.

	Pre-intervention	Post-intervention	

		Early-intervention	Late-intervention
			
Total number of patients	23898	26780	30031
Total number of months	15	15	15
Total number of days	457	457	456
Mean PIA, minutes (SD)	104.3 (9.9)	77.3 (18.3)	42.2 (8.1)
Mean LOSDep, minutes (SD)	199.4 (16.8)	170.6 (25.4)	134.4 (14.5)
Mean LWBS, % (SD)	12.1 (2.2)	8.2 (3.2)	4.6 (1.7)
CTAS 1, n (%)	83 (0.3%)	56 (0.2%)	44 (0.1%)
CTAS 2, n (%)	1212 (5.1%)	1063 (4.0%)	1157 (3.9%)
CTAS 3, n (%)	7148 (29.9%)	9590 (35.8%)	8981 (29.9%)
CTAS 4, n (%)	10459 (43.8%)	13201 (49.3%)	16820 (56.0%)
CTAS 5, n (%)	1315 (5.5%)	1756 (6.6%)	1660 (5.5%)
Unspecified CTAS	3688 (15.4%)	1069 (4.0%)	1236 (4.1%)

*PIA*, time until physician initial assessment; *SD*, standard deviation; *LOSDep*, length of stay for departed patients; *LWBS*, patients who left without being seen; *CTAS*, the Canadian Triage and Acuity Scale.

* Note that patient numbers reported by CTAS level will not match patient numbers reported by patient visits to the Carbonear emergency department (ED). This discrepancy is the result of a combination of an oversight in Eastern Health’s internal reporting system, patients who visit the ED for reasons not requiring a CTAS score (e.g., intravenous specialist assessment), and omissions in paper charts.

**Table 2:** Interrupted time-series analyses showing effects of intervention on time to physician initial assessment, patient length of stay and left without being seen rates.

Emergency department efficiency indicator	Coefficient	95% confidence interval Lower band, Upper band	P value
Average physician initial assessment			
Baseline slope	0.66	−0.35, 1.66	0.20
Level change	−19.80	−33.68, −5.89	0.01*
Slope change	−2.72	−3.97, −1.48	0.001*
Average length of stay until departure from emergency department			
Baseline slope	0.63	−0.96, 2.22	0.43
Level change	−17.52	−41.63, 6.59	0.15
Slope change	−3.00	−4.87, −1.14	0.001*
Percent of patients left without being seen			
Baseline slope	0.20	−0.06, 0.46	0.13
Level change	−3.81	−6.87, −0.75	0.02*
Slope change	−0.44	−0.73, −0.15	0.0014*

* P value < 0.05.

**Table 3:** Three-segment statistical model showing a statistically non-significant level change in both early intervention and late interventions.

Emergency department efficiency indicator	Coefficient	95% confidence interval Lower band, Upper band	P value
Average physician initial assessment			
Baseline slope	0.66	−0.30, 1.61	0.17
EI: Level change	−5.59	−13.99, 2.81	0.19
EI: Slope change	−4.45	−5.59, −3.32	0.001*
LI: Level change	−13.99	−23.35, −4.63	0.001*
LI: Slope change	5.12	4.19, 6.06	0.001*
EI: Linear trend	−3.80	−4.45, −3.15	0.001*
LI: Linear trend	1.32	0.60, 2.05	0.001*
Average length of stay until departure from emergency department			
Baseline slope	0.63	−0.58, 1.84	0.30
EI: Level change	1.93	−15.35, 19.20	0.82
EI: Slope change	−5.74	−7.69, −3.79	0.001*
LI: Level change	0.43	−19.60, 20.45	0.97
LI: Slope change	5.71	3.48, 7.95	0.001*
EI: Linear trend	−5.11	−6.64, −3.58	0.001*
LI: Linear trend	0.60	−0.92, 2.12	0.43
Percent of patients left without being seen			
Baseline slope	0.20	−0.09, 0.49	0.17
EI: Level change	−1.42	−4.22, 1.37	0.31
EI: Slope change	−0.78	−1.14, −0.43	0.001*
LI: Level change	0.41	−2.23, 3.04	0.76
LI: Slope change	0.68	0.41, 0.95	0.001*
EI: Linear trend	−0.58	−0.77, −0.40	0.001*
LI: Linear trend	0.09	−0.12, 0.31	0.38

*EI*, early intervention (October 1, 2014 to December 31, 2015); *LI*, late intervention (January 1, 2016 to March 31, 2017).

* P value < 0.05.

**Table 4:** Number of visits per month and time to physician initial assessment and left without being seen rates.

Emergency department efficiency indicator	Coefficient	95% confidence interval Lower band, Upper band	P value
Average physician initial assessment			
Visits	0.02	0.00, 0.04	0.05*
Baseline slope	0.76	0.07, 1.46	0.03*
EI: Level change	−6.16	−13.39, 1.07	0.09
EI: Slope change	−5.19	−6.60, −3.78	0.001*
LI: Level change	−11.69	−22.85, −0.52	0.04*
LI: Slope change	5.48	4.30, 6.65	0.001*
EI: Linear trend	−4.43	−5.55, −3.30	0.001*
LI: Linear trend	1.05	0.18, 1.93	0.02*
Average length of stay until departure from emergency department			
Visits	0.00	−0.04, 0.04	0.96
Baseline slope	0.64	−0.62, 1.89	0.31
EI: Level change	1.90	−15.56, 19.36	0.83
EI: Slope change	−5.77	−8.42, −3.12	0.001*
LI: Level change	0.53	−22.67, 23.72	0.96
LI: Slope change	5.73	3.46, 8.00	0.001*
EI: Linear trend	−5.14	−7.25, −3.03	0.001*
LI: Linear trend	0.59	−1.22, 2.40	0.51
Percent of patients left without being seen			
Visits	0.01	0.00, 0.01	0.04*
Baseline slope	0.23	0.02, 0.44	0.03*
EI: Level change	−1.57	−3.93, 0.80	0.19
EI: Slope change	−0.97	−1.37, −0.57	0.001*
LI: Level change	1.00	−2.16, 4.16	0.53
LI: Slope change	0.77	0.44, 1.09	0.001*
EI: Linear trend	−0.74	−1.05, −0.43	0.001*
LI: Linear trend	0.03	−0.23, 0.28	0.84

*EI*, early intervention (October 1, 2014 to December 31, 2015); *LI*, late intervention (January 1, 2016 to March 31, 2017).

* P Value < 0.05.
