# Supplementary material for: SurgeCon: Priming a Community Emergency Department for Patient Flow Management
Source: West J Emerg Med. 2019 Jul 5;20(4):654–65. doi: 10.5811/westjem.2019.5.42027 (PMC6625694; doi:10.5811/westjem.2019.5.42027)
Supplement: Supplementary file 1 [file wjem-20-654-s001.docx]

**Appendix I.** SurgeCon interrupted time series (ITS) model.

The single-group ITS model (multiple treatments: early and late interventions, each 15 months) is defined as follows:

(1) $Y_{t}=\beta_{0}+\beta_{1}T_{t}+\beta_{2}{EX}_{t}+\beta_{3}{EX}_{t}T_{t}+\beta_{4}{LX}_{t}+\beta_{5}{LX}_{t}T_{t}+\epsilon_{t}$

*where*

- $Y_{t}$ is the outcome at time *t.* In this model, we have three outcomes; PIA, LOSDep, and LWBS,
- $T_{t}$ represents the time since the beginning of the study,
- ${EX}_{t}$ is the dummy variable indicating the early intervention (pre-intervention period=0, otherwise=1),
- ${LX}_{t}$ is the dummy variable indicating the late intervention (pre-intervention period=0, otherwise=1),
- $EX_{t}T_{t}$ and $LX_{t}T_{t}$ indicate the interaction term between time and intervention.

Of the six parameters in the model (1), $\beta_{0}$ is the intercept when T=0. $\beta_{1}$ denotes the slope of time or change in outcome as the time changes until the introduction of the intervention. $\beta_{2}$ represents the level change of outcome following the intervention. $\beta_{3}$ indicates the difference between pre-intervention and post-intervention slopes of the outcome. In short, $\beta_{2}$ and $\beta_{3}$ represent changes in level and trend (slope) of outcome over the early-intervention period, respectively. Similarly, $\beta_{4}$ and $\beta_{5}$ refer to changes in level and trend (slope) of outcome during the late-intervention period.

Therefore, we look at significant p-values (p<0.05) in $\beta_{2}$ and $\beta_{4}$ for an immediate intervention effect, and $\beta_{3}\mathrm{and}\beta_{5}$ to see the impact of intervention effect over time.

To correct for autocorrelation, we adjusted the estimated model in terms of serial correlation, examining the autocorrelation structure using a Cumby-Huizinga test to ensure the fitted model is accurate,^[[1]](#footnote-1)^

1. - The values for *Cumby-Huizinga test* are available from the authors upon request. [↑](#footnote-ref-1)
