# Supplementary material for: SurgeCon: Priming a Community Emergency Department for Patient Flow Management
Source: West J Emerg Med. 2019 Jul 5;20(4):654–65. doi: 10.5811/westjem.2019.5.42027 (PMC6625694; doi:10.5811/westjem.2019.5.42027)
Supplement: Supplementary file 2 [file wjem-20-654-s002.docx]

**Appendix II.** The SurgeCon Algorithm.


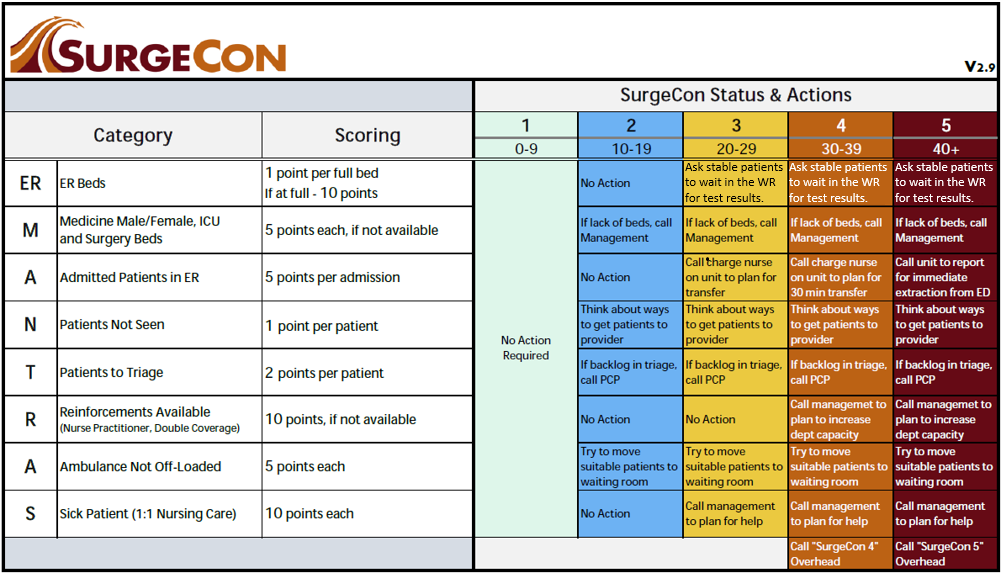


*PCP,* Primary Care Paramedic (Refers to a Paramedic trained in emergency triage).

*WR*, Waiting Room.
